# Targeted Prevention of Common Mental Health Disorders in University Students: Randomised Controlled Trial of a Transdiagnostic Trait-Focused Web-Based Intervention

**DOI:** 10.1371/journal.pone.0093621

**Published:** 2014-04-15

**Authors:** Peter Musiat, Patricia Conrod, Janet Treasure, Andre Tylee, Chris Williams, Ulrike Schmidt

**Affiliations:** 1 Department of Psychology, King's College London, Institute of Psychiatry, London, United Kingdom; 2 Centre hospitalier universitaire Sainte-Justine, Université de Montréal, Montréal, Canada; 3 Department of Psychological Medicine, King's College London, Institute of Psychiatry, London, United Kingdom; 4 Department of Health Service and Population Research, King's College London, Institute of Psychiatry, London, United Kingdom; 5 Institute of Health and Wellbeing, University of Glasgow, Glasgow, United Kingdom; United (Osaka U, Kanazawa U, Hamamatsu U Sch Med, Chiba U and Fukui U) Graduate School of Child Developmen, Japan

## Abstract

**Background:**

A large proportion of university students show symptoms of common mental disorders, such as depression, anxiety, substance use disorders and eating disorders. Novel interventions are required that target underlying factors of multiple disorders.

**Aims:**

To evaluate the efficacy of a transdiagnostic trait-focused web-based intervention aimed at reducing symptoms of common mental disorders in university students.

**Method:**

Students were recruited online (*n* = 1047, age: *M* = 21.8, *SD* = 4.2) and categorised into being at high or low risk for mental disorders based on their personality traits. Participants were allocated to a cognitive-behavioural trait-focused (*n* = 519) or a control intervention (*n* = 528) using computerised simple randomisation. Both interventions were fully automated and delivered online (trial registration: ISRCTN14342225). Participants were blinded and outcomes were self-assessed at baseline, at 6 weeks and at 12 weeks after registration. Primary outcomes were current depression and anxiety, assessed on the Patient Health Questionnaire (PHQ9) and Generalised Anxiety Disorder Scale (GAD7). Secondary outcome measures focused on alcohol use, disordered eating, and other outcomes.

**Results:**

Students at high risk were successfully identified using personality indicators and reported poorer mental health. A total of 520 students completed the 6-week follow-up and 401 students completed the 12-week follow-up. Attrition was high across intervention groups, but comparable to other web-based interventions. Mixed effects analyses revealed that at 12-week follow up the trait-focused intervention reduced depression scores by 3.58 (*p*<.001, 95%CI [5.19, 1.98]) and anxiety scores by 2.87 (*p* = .018, 95%CI [1.31, 4.43]) in students at high risk. In high-risk students, between group effect sizes were 0.58 (depression) and 0.42 (anxiety). In addition, self-esteem was improved. No changes were observed regarding the use of alcohol or disordered eating.

**Conclusions:**

This study suggests that a transdiagnostic web-based intervention for university students targeting underlying personality risk factors may be a promising way of preventing common mental disorders with a low-intensity intervention.

**Trial Registration:**

ControlledTrials.com ISRCTN14342225

## Background

The transition from school to higher education is associated with a rise in the incidence of mental health problems, due to the multiple stressors and life-style changes involved [1], [2]. Compared to age-matched controls, university students have increased symptoms of mental ill health and the number of students with symptoms of mental disorders is rising [3]. A large proportion of university students reports depressive symptoms, moderate to severe levels of anxiety [4], heavy drinking [5] and symptoms of eating disorders [6]. These common mental health problems in students are disruptive to their education (e.g. in terms of impaired performance, deferment of courses, dropping out) and emotional development [7]. Prevention delivered via the internet may be a good way of engaging at risk students, as it is flexible and in keeping with young people's preferred route for seeking help [8], [9]. Although such approaches exist, to date, most preventative efforts have usually focused on single disorders such as depression and anxiety [10], [11], alcohol misuse [12], [13] or eating disorders [14], [15], typically targeting individuals with early symptoms or partial syndromes. A recent review of technology-based student mental health interventions suggested that 30% of such interventions are not effective and that some have not been specifically designed for students [16]. Comorbidity between common mental health problems is the norm [17], [18] and these disorders have overlapping aetiologies in terms of genetic and personality factors and associated information processing styles. Trait anxiety is associated with the development of depression and anxiety [19] as well as eating disorders [20]. High levels of perfectionism have been linked with depression [21], anxiety disorders [22] and eating disorders [23]. Low self-esteem is considered to be a risk factor for depression [24], substance use disorders [25] and eating disorders [26].

Thus, an approach that targets underlying vulnerability factors rather than only disorder-specific symptoms may allow for more efficient prevention of common mental health problems [27], [28]. We developed a transdiagnostic trait-focused web-based intervention targeting personality risk factors with the aim to prevent common mental disorders in university students. This is the first intervention to target multiple mental disorders in students by addressing shared risk factors. The study presents a randomised controlled trial of the intervention compared to an active control intervention. We firstly hypothesised that students at high risk for developing common mental disorders can be identified using personality variables. Our second and main hypothesis was that the trait-focused intervention would reduce depression and anxiety in students at high risk. Finally, we hypothesised that the intervention would improve other outcomes including disorder specific (alcohol use and disordered eating) and personality variables (self-esteem, perfectionism) in students at high risk.

## Method

### Participants

Undergraduate and postgraduate students aged 18 or older were recruited between October 2011 and January 2012 via email circulars from two major London universities, inviting them to participate in a study on personality strengths and weaknesses. The recruitment email was sent to approximately 95,000 students. There were no exclusion criteria. Given the online recruitment procedure and the age of participants, it was assumed that students would have sufficient internet literacy to participate. Students were offered a £15 voucher for an online shopping site by way of thanks for their time upon completion of all assessments. Ethical approval for the study was given by the King's College London Psychiatry, Midwifery and Nursing Research Ethics Sub-committee (REF PNM 10/11-101) and the University College London Research Ethics Committee (no reference number provided).

### Design and procedure

The protocol for this trial and supporting CONSORT checklist are available as supporting information; see Checklist S1 and Protocol S1. This study was a parallel-group, placebo-controlled, blinded randomised controlled trial. To participate, students had to register and create an online account on the project website, after providing informed consent. All participants involved in this study provided informed consent by clicking a checkbox on the online information sheet, as there was no personal contact with potential participants. This procedure was approved by the involved ethics committees (see above) and access to the website was only granted after providing consent. Participants had to provide a valid email address, information about their age, course, university, ethnicity, height and weight. Upon completion of the baseline assessment, students received access to the fully-automated web-based intervention. An active control intervention was included and participants were blinded with regard to the intervention received. The randomisation sequence was generated within the web-based intervention, following a simple randomisation principle. Given that data collection was conducted online, no assessor bias was present. Immediate summative and normative feedback was provided with respect to each questionnaire following guidelines for personalised computerised feedback [29]. Six and 12 weeks after completing the baseline assessment (between January 2012 and May 2012), participants received an email reminder for the follow-up assessments. The final follow-up assessment could only be completed if the 6-week follow-up had been completed. Participants could contact the principal investigator only for technical issues and received no other type of personal support. This trial was retrospectively registered, as the authors were unaware of the journal requirements. No changes to the protocol were made. The authors confirm that all ongoing and related trials for this intervention are registered.

### Interventions

#### Transdiagnostic trait-focused online intervention

This was called “PLUS” (Personality and Living of University Students) and was described as an online resource for students to learn more about their strengths and weaknesses, and how to deal with the challenges of student life. The intervention consisted of five cognitive behavioural modules addressing a range of cognitive and behavioural interventions that aimed to help users identify strengths, and build on their weaker coping strategies. Underlying the content, a simple to understand CBT self-assessment model using the Five Areas approach was used [30]. This model has been used in a number of published book and online self-help resources and has also been found to be effective in previous RCTs for depression [31], medically unexplained symptoms [32], and eating disorders [33]. An introductory module explains basic cognitive behavioural principles, such as the connection between thoughts, feelings, physical sensations and behaviour. The remaining modules target low self-esteem, trait anxiety and worry, perfectionism, and emotional dysregulation. Participants could complete modules in any order. Each module focuses on the potentially negative impact of personality traits on certain aspects of life and how students can overcome this. Hence, the modules do not aim to modify personality risk factors, but were designed to help students recognize and reduce unhelpful behaviours and thoughts resulting from certain personality risk factors. Table 1 outlines the content of the intervention modules. The modules were chosen based on a review of risk factors of common mental disorders in students, a review of existing intervention for the students, as well as a series of focus groups with students. Each module content was developed by the authors of this paper and in collaboration with other clinical colleagues and students (as potential consumers of the intervention). Modules were text-based and included photographic and other illustrations throughout. Completion of each module approximately takes 20 to 40 minutes.

**Table 1:** Outline of trait-focused intervention content.

Module	Content
Introduction	Introduction to cognitive-behavioral principles
	5 areas assessment model
	impact of personality on behaviour
Perfectionism	Positive and negative aspects of perfectionism
	Unhelpful patterns of perfectionistic thinking
	Identifying and challenging perfectionistic thoughts and behaviour
Self-esteem	Sources of self-esteem
	Unhelpful behaviour as a result of low self-esteem
	Strategies for overcoming low self-esteem
Anxiety and worry	Behavioural consequences of trait-anxiety and worry
	Strategies for identifying and reducing the impact of trait-anxiety and worry
Dealing with difficult emotions	Function and consequences of emotions
	Emotional instability (Neuroticism) and unhelpful behaviour
	Strategies for emotional regulation

#### Control Intervention

This consisted of three online modules addressing relevant topics of student life, namely how to find accommodation, how to live on a tight budget, and study skills (time management, working with scientific texts). Students could complete these modules at any time. For that reason, the second time of measurement was chosen as the primary time point of comparison. The choice of modules for the control intervention was based on results of a series of focus groups with students. In these focus groups, students were asked to identify major challenges of student living when they entered higher education and throughout their studies. Student identified social challenges (e.g. meeting new people), practical challenges (e.g. money, housing), academic challenges (e.g. workload) and adjustment challenges (e.g. increased responsibility). Based on these findings, the control intervention was designed to support students with the practical and academic challenges. Modules were text-based and included photographic and other illustrations throughout. Completion of each module in the control group takes approximately 15 to 30 minutes.

### Measures

#### Personality trait measures

To assess whether a participant was at high risk for developing common mental disorders, four personality traits (Neuroticism, Concern over Mistakes, Doubts about Actions and Hopelessness) derived from different measures were used in a logistic regression model to identify students at high risk. This regression model is based on results from a previous study, in which students (N = 425) were assessed on a range of personality and mental health variables. Using cluster analysis, students were grouped into those reporting sub-threshold mental health problems and those who reported no symptoms. A logistic regression model was developed that allows identifying the risk status of an individual based on their personality. The personality domains Neuroticism, Concern over Mistakes, Doubts about Actions and Hopelessness best predicted risk and correctly classified 86.6% of students.

Neuroticism is associated with the development of depression and anxiety [19] as well as eating disorders [20]. To assess neuroticism, the short version of the revised NEO Five-Factor Personality Inventory (NEO-FFI) [34] was used, which assesses personality on the domains: Neuroticism, Extraversion, Openness, Agreeableness and Conscientiousness. The test authors have reported internal consistencies with Cronbach's α ranging from .86 to .92 and test-retest reliabilities of .79 to .83 [34].

To assess Concern over Mistakes and Doubts about Actions, the Frost Multidimensional Perfectionism Scale (FMPS) was used. This self-report measure assesses perfectionism on the dimensions: Concerns over Mistakes, Personal Standards, Parental Expectations, Parental Criticism, Doubts about Actions, and Organization [35]. High levels of perfectionism have been linked with depression [21], anxiety disorders [22] and eating disorders [23]. In the present study, only the subscales Concern over Mistakes, Personal Standards and Doubts about Action were included, as these are the most robust facets of the questionnaire [36]. The original authors of this questionnaire demonstrated a good reliability of the FMPS. Internal consistencies (Cronbach's α) for the subscales ranged from .77 to .93 and an overall internal consistency of .90 was reported.

The Substance Use Risk Profile Scale (SURPS) is a self-report measure assessing four personality profiles with different motivations for the use of alcohol and drugs [37] and was used in this study to assess Hopelessness (H). The Hopelessness scale assesses to what extent the individual habitually feels unhappy or negative towards the future. Hopelessness play an important role in the development of depression and is associated with suicidal ideation [38]. In a study with 462 undergraduates by Woicik et al. [37] an internal consistency of .86, as well as a test-retest reliability of .75 for the hopelessness subscale was reported.

#### Outcome measures

The Patient Health Questionnaire 9 (PHQ-9) [39]: This commonly used 9-item self-report questionnaire assesses depressive symptoms over the previous two weeks. The PHQ-9 has a high internal consistency of .89 and a good test-retest reliability of .84 [39]. Generalised Anxiety Disorder scale (GAD-7) [40]: This seven-item-scale assesses the frequency of anxiety symptoms within the past two weeks. The GAD-7 has excellent internal consistency (Cronbach's α = .92) and a good test-retest reliability of .83 [40]. Alcohol Use Disorders Identification Test (AUDIT): This self-report measure by the World Health Organization [41], was used to assess presence of harmful drinking patterns over the previous year. The reliability of the AUDIT in a student sample was investigated by Fleming et al. [42]. They reported the internal consistency (Cronbach's α) as .80 and a sensitivity of 84% at a cut-off of 11. The test-retest reliability of the AUDIT is reported as .86 by the authors of the measure [41]. The Eating Disorders Diagnostics Scale (EDDS) [43] was used to assess symptoms of eating disorders over the previous three months. A symptom composite was obtained by summing the responses of all items apart from those referring to weight, height and the use of oral contraceptives. The authors of the measure reported a one-week test-retest reliability of r = .87 for the symptom composite. Perfectionism was assessed using the Frost Multidimensional Perfectionism Scale (see above). The Rosenberg Self-Esteem Scale (RSES) [44] was used to assess self-esteem. In a study with university students, a test-retest reliability of .84 was observed after a period of four weeks [45]. The WHOQOL-BREF [46] is a self-report measure which assesses quality of life on four different domains: physical health, psychological health, social relationships, and environment. In the original validation study, good internal consistencies for the domains were reported with Cronbach's α ranging from .66 (social relationships) to .84 (psychological health). The test-retest reliability in the same study was reported to range from .66 for physical health to 0.87 for environment [47]. The environment scale was omitted from the questionnaire in this study, as it assesses the availability of different resources (e.g. financial, health care), which would not be influenced by the intervention.

At the 12-week follow-up assessment, students were asked to complete an optional questionnaire on the satisfaction with the online intervention. This questionnaire included visual analogues scale on helpfulness of the automated feedback, the helpfulness of the intervention, and the ease of use and design of the website.

### Statistical analyses

To detect students at high risk for developing common mental disorders, a binary logistic regression model was used, classifying students according to their personality. Given the transdiagnostic character of the intervention, two primary outcomes were used in this study: PHQ-9 and GAD-7 scores. Secondary outcomes were self-esteem, perfectionism (FMPS), alcohol misuse (AUDIT), and disordered eating (EDDS). Independent sample *t*-tests were used to compare students at high risk and low risk on primary and secondary outcomes.

Linear mixed models were used to evaluate the efficacy of the intervention. Each outcome was analysed by a separate model. Students' risk status and assessment time were included as predictors (independent variables) into the model, as well as a two-way interaction term for intervention group × assessment time and a three-way interaction term for intervention group × assessment time × risk status. A random intercept for each subject was included in the model. Predictors were entered untransformed and an unstructured covariance matrix and maximum likelihood estimation were used. Using logistic regression models, we assessed whether any baseline variable predicts missingness of data, as linear mixed models only provide valid estimates if data is missing at random. Contrasts analysis was carried out to compare baseline scores against results from the 12-week follow-up and to compare both interventions at 12-week follow-up. Analyses were carried out using the SPSS statistical software package (version 20). An a priori sample size calculation was conducted: To detect a medium effect (*f* ^2^ = 0.15) in students at high risk in a linear regression model at a power of 0.95 and a significance level of 5%, and assuming a correlation between measures of *r* = .5, a sample size of *N* = 196 is required. To account for the fact that approximately only 20% of students are at high risk for developing mental health problems [4] and an estimated dropout rate of 30%, the total sample size required is *N* = 700.

## Results

### Enrolment and Students at High Risk

A total of 1141 students created an online profile with the website. Table 2 shows demographic characteristics of the entire sample and the randomisation groups at baseline. Intervention groups were tested for differences on demographic and baseline variables and no significant differences were found. Of those students, 1047 completed all baseline measures and were randomised to an intervention. The results of the logistic regression suggested that 17.4% of students who completed the baseline assessment were classified as high risk for the development of common mental disorders using personality variables and the logistic regression model. Table 3 shows the difference between students at high and low risk on all baseline measures. Given the number of variables, a Bonferroni correction was applied lowering the level of significance to .0045%.

**Table 2:** Demographic Characteristics of Participants at Baseline.

Baseline Characteristic	Entire sample	Trait-focused	Active control
Age: Range (*Mdn*)	18–57 (21)	18–52 (21)	18–57 (21)
Sex: *n* (*%*)			
Female	804 (70.5)	372 (71.7)	358 (69.6)
Male	337 (29.5)	147 (28.3)	156 (30.4)
BMI: *M* (*SD*)	22.0 (3.7)	22.0 (3.6)	21.9 (3.8)
Year of studies: Range (*Mdn*)	1–6 (1)	1–6 (1)	1–6 (1)
Ethnicity: *n* (*%*)			
British Asian	87 (8.4)	44 (8.5)	43 (8.4)
Asian other	145 (14.0)	68 (13.1)	77 (15.0)
Black British	10 (1.0)	3 (0.6)	7 (1.4)
Black other	1 (0.1)	1 (0.2)	0 (0.0)
Other	77 (7.5)	36 (6.9)	41 (8.0)
White British	503 (48.7)	257 (49.5)	246 (47.9)
White other	210 (20.3)	110 (21.2)	100 (19.5)
Marital status: *n* (*%*)			
Divorced	2 (0.2)	0 (0.0)	2 (0.4)
Living together	110 (10.6)	67 (12.9)	43 (8.4)
Married	35 (3.4)	18 (3.5)	17 (3.3)
Separated	3 (0.3)	2 (0.4)	1 (0.2)
Single	883 (85.5)	432 (83.2)	451 (87.7)
Housing situation: *n* (*%*)			
Living alone	92 (8.9)	52 (10.0)	40 (7.8)
Shared accommodation	508 (49.2)	254 (48.9)	254 (49.4)
Student halls	302 (29.2)	141 (27.2)	161 (31.3)
With parents	131 (12.7)	72 (13.9)	59 (11.5)

**Table 3:** Difference at baseline between students at high risk and low risk.

Scale	Risk		*M_diff_* [95% *CI*]	*p*
	Low (*N* = 859) *M* (*SD*)	High (*N* = 181) *M* (*SD*)		
Depression	5.31 (4.43)	14.57 (6.02)	−9.26 [−10.20, −8.32]	<.001
Generalised anxiety	3.89 (3.94)	11.43 (5.26)	−7.54 [−8.36, −6.71]	<.001
Alcohol use	6.22 (4.96)	7.16 (6.22)	−0.93 [−1.91, 0.04]	.061
Disordered eating	5.26 (3.44)	8.36 (3.78)	−3.10 [−3.69, −2.52]	<.001
Concern over mistakes	23.98 (7.04)	34.29 (6.68)	−10.31 [−11.43, −9.19]	<.001^a^
Personal standards	24.93 (4.98)	26.38 (5.48)	−1.44 [−2.31, −0.57]	.001
Doubts about actions	10.59 (3.32)	15.48 (2.89)	−4.89 [−5.37, −4.41]	<.001^a^
Self-esteem	20.21 (5.03)	10.15 (4.34)	10.06 [9.34, 10.78]	<.001
Physical health	16.14 (2.13)	13.29 (2.62)	2.85 [2.44, 3.26]	<.001
Psychological health	14.31 (2.61)	9.04 (2.68)	5.27 [4.85, 5.70]	<.001
Social relationships	14.01 (3.41)	10.66 (3.71)	3.35 [2.78, 3.91]	<.001

a these variable were used in the logistic regression model to classify students and show significant differences per definition.

Using a logistic regression model developed in a previous study (Musiat et al., in preparation), students were grouped into high and low risk for developing mental health problems. In this model, students with a combination of high levels of Neuroticism (NEO-FFI), Concern over Mistakes (FMPS), Doubts about Actions (FMPS) and Hopelessness (SURPS) are considered at high risk. At baseline, students at high risk were found to report higher levels of depression and anxiety as well as higher levels of disordered eating, higher perfectionism, lower self-esteem and lower quality of life on all assessed domains. No differences between the groups at baseline were found on the AUDIT score, suggesting that the frequency of drinking and consequences related to drinking use are comparable between high and low risk students. These findings suggest that students classified as high risk according to their personality show significantly poorer mental health on several domains and report symptoms of some but not all common mental disorders.

### Attrition

**Figure 1:**
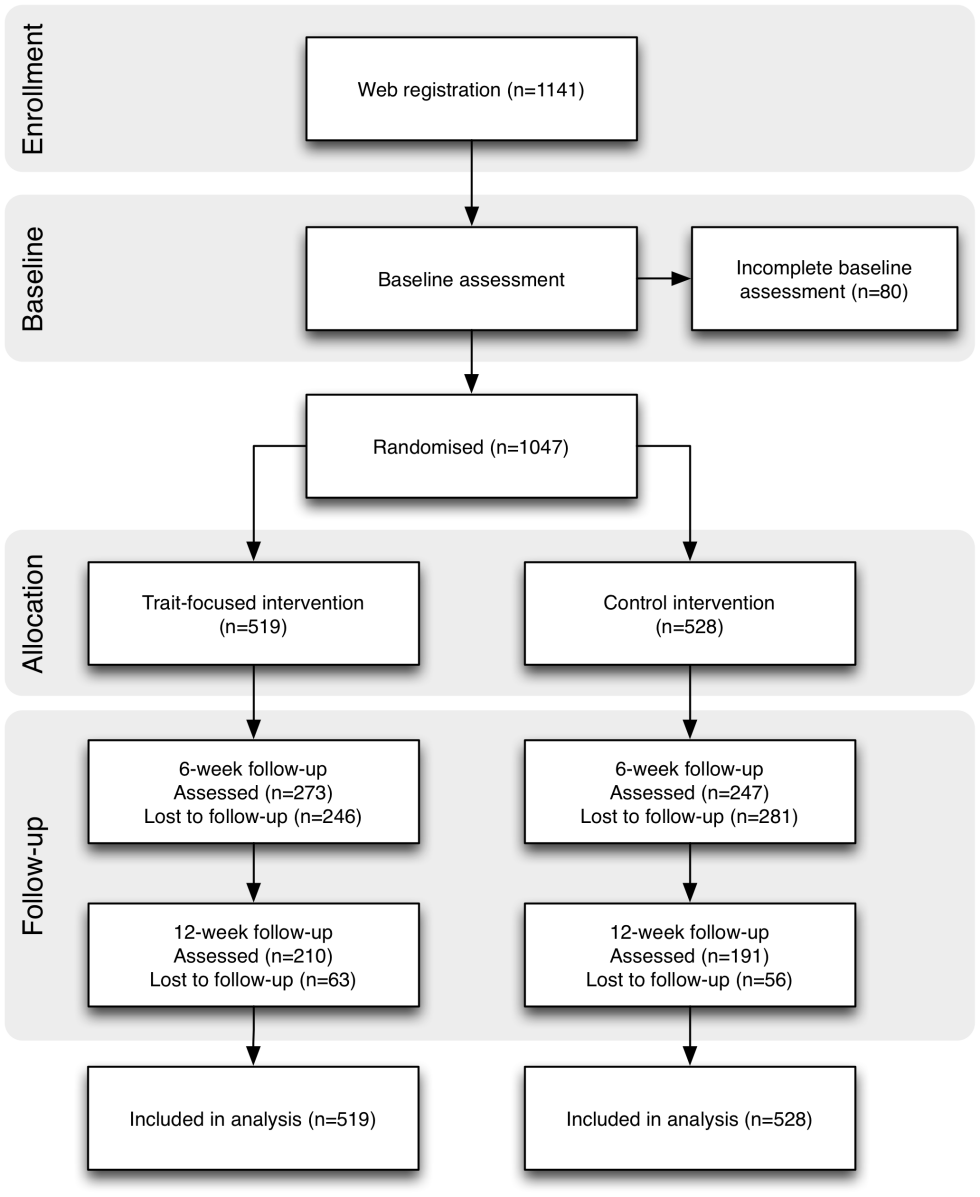
Participant flow through the trial.

Figure 1 shows the participant flow through the study. Half of the students (50.3%) dropped from the trial at the 6-week follow-up and a further 11.4% at 12-week follow up. Total attrition was 61.7% by 12 weeks. Therefore, 520 students completed the 6-weeks follow up and 401 completed the 12-week follow up assessment. To test whether or not students with higher psychopathology were more likely to drop out, a chi-square test was performed comparing the number of students at risk between students who dropped out and those, who did not. No significant differences in terms of the proportion of students at risk were found between dropouts at T_1_ or T_2_ and completers. No differences between the intervention and control group were observed with regard to dropout.

Results from the logistic regression suggested that only physical health as assessed with the WHOQOL and impulsivity as assessed with the SURPS significantly predicted missingness/drop-out at 6-week or 12-week follow-up. Students at high risk for developing common mental disorders differed significantly on these variables from students at low risk. As the risk status was included as a predictor in the linear mixed model and shares variance with these two variables, physical health and impulsivity were not included as predictors in the model.

Summary data on the completion of the online modules revealed that at 12-week follow-up in the control group, 81% of students had fully completed an online module after starting it. In contrast, within the trait-focused intervention group, on average 47% of students had completed a module at 12-week follow-up after starting it. However, this data was not available on an individual level thus a dose-effect relationship could not be investigated.

### Intervention effects

Table 4 shows the estimated marginal means and statistics from the linear mixed model analysis and Table 5 shows the contrasts analysis comparing baseline and 12-week follow-up. A significant interaction between time, intervention group and risk status was observed for depression and generalised anxiety, suggesting that students at high risk showed greater reduction of depression and anxiety. Within group effect sizes (Cohen's *d*) for students at high risk in the intervention group were 0.61 for depression and 0.56 for generalised anxiety, between group effect sizes were 0.58 and 0.42 respectively. Although the significant interaction effect between time and intervention group suggest a general reduction of depression and anxiety regardless of risk, the contrasts analysis does not support this. No significant interactions were observed regarding the use of alcohol or disordered eating. Contrasts suggest a reduction of drinking in student at low risk, regardless of the intervention received. Significant interactions between intervention group and assessment time were observed for the perfectionism scales Concern over Mistakes and Personal Standards. Estimated means on these scales suggest that Concern of Mistakes and Personal Standards were reduced in the trait-focused intervention, whereas scores in the control group increased. The contrast analysis, however, did not support these results. No three-way interaction for Concern over mistakes, Personal standards and Doubts about actions was observed. In students at high risk, who received the trait-focused intervention, Concern over Mistakes scores were significantly reduced at 12-week follow-up. In students at high risk and who received the trait-focused intervention self-esteem was significantly increased (within group effect size *d* = 0.23, between group effect size *d* = 0.06) and there was a significant three-way interaction between assessment time, intervention group and risk status. Participants did not report any adverse events, e.g. in their feedback on the intervention or by contacting the researcher.

**Table 4:** Estimated means for intervention and control group, by risk status and results of the linear mixed models analysis.

		Trait-focused		Control		Time × group		Time × group × risk	
		Baseline	12 weeks	Baseline	12 weeks				
Variable	Risk	*M* (*SE*)	*M* (*SE*)	*M* (*SE*)	*M* (*SE*)	*F*	*p*	*F*	*p*
Primary outcomes									
Depression	High	14.64 (0.51)	11.06 (0.70)	14.50 (0.49)	14.46 (0.74)	5.11	.002	4.83	<.001
	Low	5.29 (0.23)	5.04 (0.31)	5.32 (0.23)	5.36 (0.32)				
Generalised anxiety	High	12.01 (0.45)	9.14 (0.65)	10.95 (0.44)	11.26 (0.69)	5.25	.001	2.75	.018
	Low	3.89 (0.20)	3.47 (0.29)	3.89 (0.21)	4.19 (0.30)				
Secondary outcomes									
Alcohol use	High	7.89 (0.56)	7.47 (0.59)	6.48 (0.54)	5.95 (0.60)	1.29	.277	0.99	.422
	Low	6.30 (0.25)	5.42 (0.27)	6.17 (0.25)	5.51 (0.27)				
Disordered eating	High	8.71 (0.39)	7.99 (0.51)	8.07 (0.37)	7.22 (0.52)	0.39	.761	1.45	.204
	Low	5.13 (0.17)	4.99 (0.22)	5.28 (0.17)	4.92 (0.23)				
Concern over mistakes	High	34.71 (0.75)	32.28 (0.93)	33.90 (0.72)	34.22 (0.97)	3.04	.029	2.13	.060
	Low	24.07 (0.34)	23.85 (0.42)	23.90 (0.34)	24.40 (0.43)				
Personal standards	High	27.21 (0.54)	26.45 (0.65)	25.61 (0.52)	26.34 (0.68)	2.68	.046	1.88	.097
	Low	24.67 (0.24)	24.10 (0.29)	25.20 (0.24)	24.83 (0.30)				
Doubts about actions	High	15.72 (0.35)	14.91 (0.48)	15.25 (0.34)	15.77 (0.50)	2.38	.068	1.61	.155
	Low	10.69 (0.16)	10.98 (0.21)	10.48 (0.16)	10.99 (0.22)				
Self-esteem	High	9.23 (0.53)	10.67 (0.68)	11.01 (0.51)	11.09 (0.71)	2.24	.082	3.10	.009
	Low	20.18 (0.24)	19.82 (0.30)	20.25 (0.24)	19.90 (0.31)				

**Table 5:** Analysis of contrasts between baseline and 12-week follow-up.

		Trait-focused		Control	
Variable	Risk	*Mean difference* [95% CI]^a^	*p*	*Mean difference* [95% CI]^a^	*p*
Primary outcomes					
Depression	High	3.58 [1.98, 5.19]	<.001	0.04 [−1.68, 1.75]	1.000
	Low	0.25 [−0.46, 0.97]	1.000	−0.03 [−0.78, 0.71]	1.000
Generalised anxiety	High	2.87 [1.31, 4.43]	<.001	−0.31 [−1.97, 1.35]	1.000
	Low	0.43 [−0.26, 1.11]	.415	−0.31 [−1.02, 0.41]	.920
Secondary outcomes					
Alcohol use	High	0.42 [−0.49, 1.33]	.798	0.53 [−0.44, 1.50]	.575
	Low	0.88 [0.47, 1.30]	<.001	0.66 [0.24, 1.09]	.001
Disordered eating	High	0.72 [−0.17, 1.60]	.156	0.85 [−0.12, 1.82]	.106
	Low	0.15 [−0.25, 0.54]	1.000	0.37 [−0.04, 0.77]	.090
Concern over mistakes	High	2.42 [0.60, 4.25]	.005	−0.32 [−2.29, 1.65]	1.000
	Low	0.22 [−0.59, 1.03]	1.000	−0.50 [−1.35, 0.34]	.459
Personal standards	High	0.75 [−0.54, 2.05]	.491	−0.74 [−2.13, 0.66]	.617
	Low	0.57 [−0.01, 1.15]	.053	0.37 [−0.23, 0.97]	.403
Doubts about actions	High	0.81 [−0.19, 1.82]	.158	−0.52 [−1.60, 0.56]	.752
	Low	−0.28 [−0.73, 0.17]	.392	−0.51 [−0.97, −0.04]	.027
Self-esteem	High	−1.44 [−2.74, −0.14]	.024	−0.08 [−1.50, 1.34]	1.000
	Low	0.36 [−0.22, 0.94]	.419	0.35 [−0.25, 0.96]	.477

a A Bonferroni correction was applied.

### Satisfaction with the intervention

Only 42% of students completed the optional user satisfaction questionnaire. With regard to the helpfulness of the automated feedback, students indicated a median score of 5 on a seven-step rating scale ranging from “not helpful at all” (1) to “very helpful” (7). To the question whether the website was difficult or easy to use, students reported a median score of 6 on a seven-step rating scale ringing from “very difficult” (1) to “very easy” (7). To what extent students liked the design of the website, a median score of 5 on a seven-step rating scale ranging from “not at all” (1) to “very much” (7) was reported. There were no significant differences with regard to satisfaction ratings between the intervention conditions.

## Discussion

This study aimed to investigate the efficacy of a transdiagnostic trait-focused web-based intervention aimed at preventing common mental disorders in students. It was hypothesised that students at high risk for developing common mental disorders could be identified according to their levels of trait anxiety, perfectionism and hopelessness. This hypothesis was confirmed and students identified by the logistic regression model showed significantly poorer mental health compared to students at low risk. Our main hypothesis was that the intervention would reduce current anxiety and depression in students at high risk and this hypothesis was confirmed. With regard to secondary outcomes, no reduction of alcohol use, disordered eating or on the perfectionism subscales of the FMPS was observed. Although contrasts suggested a slight reduction of Concern over Mistakes in students at high risk, the mixed model analysis did not support this. As hypothesised, self-esteem was increased by the trait-focused intervention in students at high risk. In summary, our main hypothesis was confirmed, whereas only partial support was found for the third hypothesis.

This study evaluated the first trait-focused web-based intervention designed to address common mental disorders in students and hence presented an innovative intervention with the potential of large impact. Although prevention programs targeting mental health in students exist, most target only one disorder and focus on a subsample of students who exhibit symptoms. This study provides further support for the idea of preventing mental disorders by targeting underlying vulnerability factors, such as trait-anxiety and emotional dysregulation [28]. Similar to the study by Kenardy et al. [48], depression and anxiety could be reduced in students at high risk for developing mental disorders. Two main characteristics of the present study set it apart from previous research. Firstly, students at high risk were identified according to their personality characteristics and not by symptomatology. Secondly, the intervention in this study targeted several common mental disorders, including depression, anxiety, substance use and eating disorders. Given that at baseline, the differences between students at high risk and students at low risk with regard to depression and anxiety were much larger than on any other of the assessed variables, it is likely that changes in these domains are easier to achieve than in others, resulting in larger effect sizes. The intervention also improved self-esteem in students at high risk. Very few interventions targeting self-esteem in university students exist and often only as secondary outcomes in the prevention of e.g. eating disorders [49], [50]. Similar to this study, the effects in such studies were often small [51]. No effects were observed with regard to perfectionism. Although the role of perfectionism in university students has been discussed extensively in relation to loneliness, shyness and self-esteem [52]. or adjustment and mental health problems [53], interventions targeting perfectionism are rare and often produce only small effects [54]. Given the relatively high stability of perfectionism across age [55], it is possible that perfectionism is difficult to modify with a brief web-based intervention.

Using the personality variables Neuroticism, Concern over Mistakes, Doubts about Actions and Hopelessness, students at high risk for developing common mental disorders were identified in a logistic regression model. These findings support previous evidence on student mental health, suggesting that approximately 20% of students are affected by symptoms of mental disorders, such as low mood and anxiety [4]. It is interesting to note, that students detected in this model show various symptoms of mental disorders including disordered eating and low self-esteem. Although we do not argue that personality variables have greater diagnostic value with regard to detecting student at high risk for common mental disorders, we think that they could be a useful alternative to a symptom-focused assessment, particularly in the context of prevention interventions. In addition, the inclusion of personality risk factors addresses the limitation of diagnostic criteria [28] and the overlap in aetiologies of common mental disorders [19]. Instead of promoting the study in the context of mental health, we introduced it to students as a study on personality, strengths and weaknesses, resulting in great interest from students and the large sample size. Recruitment attracted more female then male students. Although both recruitment sites had more female (61% and 54%) than male students during the recruitment period, the proportion of female students in this study was considerably higher (71%). It is possible that the study was more appealing to female student, but may also reflect the gender differences in the prevalence and help-seeking behaviour of common mental disorders [56].

Differences found with regard to module completion can be attributed to numerous factors. First, modules in the control group were likely to appeal to a majority of students, whereas modules in the trait-focused group were more relevant to students at high risk. In addition, in the trait-focused group, student could download PDF copies of the modules therefore eliminating the need to complete them online. There were also fewer and shorter modules in the control group. However, it has to be noted that despite the differences in length and content between the trait-focused intervention and the control intervention modules, there was no difference in dropout rates between the intervention groups and students rated the content of both interventions as helpful. In this study, students could complete the intervention modules in any order. It allowed students to flexibly access the resources they considered potentially useful for them, guided by the feedback they received in the baseline assessment. However, this requires a high degree of motivation and is likely to have contributed to the low rates of module completion, as there was no prescribed sequence for completing the intervention.

This study did not include a personal support component. Although personal support in computerised interventions is often associated with higher efficacy and lower dropout [57], this intervention was designed as a pure self-help resource that does not require personal contact or support. Thus, the intervention could also be widely implemented without the need for creating additional infrastructure to accommodate more users.

### Strengths and limitations

As the design was a randomised controlled trial (RCT), the evaluation was controlled for possible confounders allowing a valid evaluation of the efficacy. A large sample size was used and the intervention was administered to all students regardless of their risk status, which made it possible to examine whether the intervention can be used universally. This sets it apart from other studies, which focused on high-risk individuals only. Another strength of this study was the inclusion of an active control group, which is in accordance with the guidance on the development of complex interventions by the Medical Research Council [58]. Feedback from students on the perceived helpfulness was positive for both interventions. This suggests that despite the differences in length, content and format, the active control was a credible intervention. In addition, although the control intervention addressed issues that were identified by students, it produced no improvements with regard to students' early symptoms of mental health problems, which has important implications for the prevention of common mental health disorders in students, namely that addressing these common stressors, as is often done within university services, does on its own not appear to be effective in improving mental health in students.

One of the main limitations of this study is the short follow-up period. It is possible that over time more students would have made use of the student modules and that the techniques taught in the modules take some time to induce change. However, this choice was made based on the length of a university term. Another limitation was that feedback on the intervention was only obtained from individuals that fully completed the intervention making it likely that the information assessed was biased. In the present study using self-referring responders to advertising emails, no structured interviews or other diagnostic tools were used to assess whether students fulfilled the criteria for common mental disorders. Hence, it was not possible to assess how many people are affected by a disorder at clinical severity and whether the intervention worked differently in those students.

A large proportion of students who created an account with the website dropped out from this study and this limits the generalizability of the results. Although attrition in this study was high (62%), the rate is within what is to be expected in a web-based intervention trial (for a review, see Melville & Casey [59]). The results particularly compare to a recent study investigating the efficacy of MoodGYM (a web-based CBT intervention for depression), which had similar follow-up periods of six and 12 weeks and reported an overall attrition of 74% in the intervention group and only 27% in the waitlist control group [60]. These high dropout rates highlight the difficulty of engaging university students in a mental health intervention. It is possible that the length and format of the intervention did not appeal to all students and contributed to attrition. As students in this study did not necessarily suffer from a diagnosed mental disorder, their initial interest in the study diminished. The fact that a shopping voucher was offered to students potentially further contributed to the high attrition rates. Although this generated interest in a large number of students, the incentive may not have been high enough for all students to invest a considerable amount of time in the study. Thus the high attrition may somewhat be an artefact of the large number of students recruited at baseline. Both intervention conditions included personalised computerised feedback. It is possible that some students were primarily interested in the assessment and the feedback and had no intention of going through the intervention modules when signing up. After all, the study was advertised as an opportunity for students to find out about their strengths and weaknesses. In addition, adherence data unfortunately were not available on an individual level. This makes it difficult to assess whether the observed changes in the intervention group can be attributed to students accessing a particular intervention module. Although a high number of students were recruited, the dropout was much higher than anticipated. As a result, the study was underpowered to detect small effect sizes.

### Implications for future studies

The results from this study emphasise the need for mental health interventions in university students, given the high proportion of students reporting symptoms of mental disorders. This study provided preliminary support for the efficacy of a transdiagnostic trait-focused web-based targeted prevention program as an efficient way of addressing common mental disorder in university students, at least over a short time period. Furthermore, to support students in their mental health it appears not enough to provide them with material on common study-related issues, such as study skills, finance or accommodation. The study demonstrated how personality traits can be used to identify students at high risk for common mental disorders. Despite the potential of using personality traits to transdiagnostically detect risk, they also offer a non-intrusive and interesting opportunity to engage students in a health intervention. Future applications could include a personal support component to increase efficacy, reduce drop-out and to manage risk [57].

## Supporting Information

Protocol S1 **Trial protocol.**
(PDF)

Checklist S1 **CONSORT checklist.**
(DOC)

## References

[1] Fisher S, Hood B (1987) The stress of the transition to university: a longitudinal study of psychological disturbance, absent-mindedness and vulnerability to homesickness. British Journal of Psychology 78: 425–441. 3427309 10.1111/j.2044-8295.1987.tb02260.x

[2] Lu L (1994) University transition: major and minor life stressors, personality characteristics and mental health. Psychological Medicine 24: 81–87. 8208897 10.1017/s0033291700026854

[3] Royal College of Psychiatrists (2011) The Mental Health of Students in Higher Education. London: Royal College of Psychiatrists.

[4] Webb E, Ashton CH, Kelly P, Kamali F (1996) Alcohol and drug use in UK university students. Lancet 348: 922–925. 8843811 10.1016/s0140-6736(96)03410-1

[5] Johnston LD, O'Malley PM, Bachman JG, Schulenberg JE (2009) Monitoring the Future: National Survey Results on Drug Use 1975–2007. Bethesda, MD: National Institute on Drug Abuse.

[6] Eisenberg D, Nicklett EJ, Roeder K, Kirz NE (2011) Eating disorder symptoms among college students: Prevalence, persistence, correlates, and treatment-seeking. Journal of American College Health 59: 700–707. 21950250 10.1080/07448481.2010.546461 PMC3721327

[7] Brackney BE, Karabenick SA (1995) Psychopathology and Academic Performance: The Role of Motivation and Learning Strategies. Journal of Counseling Psychology 42: 456–465.

[8] Chew-Graham CA, Rogers A, Yassin N (2003) ‘I wouldn't want it on my CV or their records’: medical students' experiences of help-seeking for mental health problems. Medical Education 37: 873–880. 12974841 10.1046/j.1365-2923.2003.01627.x

[9] Oliver MI, Pearson N, Coe N, Gunnell D (2005) Help-seeking behaviour in men and women with common mental health problems: cross-sectional study. British Journal of Psychiatry 186: 297–301. 15802685 10.1192/bjp.186.4.297

[10] Kenardy J, McCafferty K, Rosa V (2006) Internet-delivered indicated prevention for anxiety disorders: Six-month follow-up. Clinical Psychologist 10: 39–42.

[11] Seligman MEP, Schulman P, Tryon AM (2007) Group prevention of depression and anxiety symptoms. Behaviour Research and Therapy 45: 1111–1126. 17074301 10.1016/j.brat.2006.09.010

[12] Paschall MJ, Antin T, Ringwalt CL, Saltz RF (2011) Evaluation of an Internet-Based Alcohol Misuse Prevention Course for College Freshmen: Findings of a Randomized Multi-Campus Trial. American Journal of Preventive Medicine 41: 300–308. 21855745 10.1016/j.amepre.2011.03.021 PMC3173258

[13] Conrod PJ, Stewart SH, Comeau N, Maclean AM (2006) Efficacy of cognitive-behavioral interventions targeting personality risk factors for youth alcohol misuse. Journal of Clinical Child and Adolescent Psychology 35: 550–563. 17007600 10.1207/s15374424jccp3504_6

[14] Stice E, Rohde P, Shaw H, Marti CN (2012) Efficacy trial of a selective prevention program targeting both eating disorder symptoms and unhealthy weight gain among female college students. Journal of Consulting and Clinical Psychology; Journal of Consulting and Clinical Psychology 80: 164. 22122289 10.1037/a0026484 PMC3265656

[15] Beintner I, Jacobi C, Taylor CB (2011) Effects of an Internet-based Prevention Programme for Eating Disorders in the USA and Germany - A Meta-analytic Review. European Eating Disorders Review. 10.1002/erv.1130 21796737

[16] Farrer L, Gulliver A, Chan JK, Batterham PJ, Reynolds J, et al (2013) Technology-based interventions for mental health in tertiary students: systematic review. Journal of Medical Internet Research 15: e101. 23711740 10.2196/jmir.2639 PMC3668609

[17] Regier DA, Farmer ME, Rae DS, Locke BZ, Keith SJ, et al (1990) Comorbidity of mental disorders with alcohol and other drug abuse. JAMA: the journal of the American Medical Association 264: 2511–2518. 2232018

[18] Bulik CM, Klump KL, Thornton L, Kaplan AS, Devlin B, et al.. (2004) Alcohol use disorder comorbidity in eating disorders: a multicenter study. Journal of Clinical Psychiatry; Journal of Clinical Psychiatry. 10.4088/jcp.v65n0718 15291691

[19] Clark LA, Watson D, Mineka S (1994) Temperament, personality, and the mood and anxiety disorders. Journal of Abnormal Psychology 103: 103–116. 8040472

[20] Ghaderi A, Scott B (2000) The big five and eating disorders: A prospective study in the general population. European Journal of Personality 14: 311–323.

[21] Shafran R, Mansell W (2001) Perfectionism and psychopathology: A review of research and treatment. Clinical Psychology Review 21: 879–906. 11497211 10.1016/s0272-7358(00)00072-6

[22] Saboonchi F, Lundh LG, Öst LG (1999) Perfectionism and self-consciousness in social phobia and panic disorder with agoraphobia. Behaviour Research and Therapy 37: 799–808. 10458045 10.1016/s0005-7967(98)00183-1

[23] Slade P (1982) Towards a functional analysis of anorexia nervosa and bulimia nervosa. British Journal of Clinical Psychology 21: 167–179. 6957251 10.1111/j.2044-8260.1982.tb00549.x

[24] Battle J (1978) Relationship between self-esteem and depression. Psychological Reports 42: 745–746. 674498 10.2466/pr0.1978.42.3.745

[25] Glindemann KE, Geller ES, Fortney JN (1999) Self-esteem and alcohol consumption: A study of college drinking behavior in a naturalistic setting. Journal of Alcohol and Drug Education 45: 60–71.

[26] Shisslak CM, Crago M, Renger R, Clark-Wagner A (1998) Self-esteem and the prevention of eating disorders. Eating Disorders 6: 105–117.

[27] Conrod PJ, O'Leary-Barrett M, Newton N, Topper L, Castellanos-Ryan N, et al.. (2013) Effectiveness of a Selective, Personality-Targeted Prevention Program for Adolescent Alcohol Use and Misuse: A Cluster Randomized Controlled Trial. JAMA Psychiatry: 1–9. 10.1001/jamapsychiatry.2013.651 23344135

[28] Brown TA, Barlow DH (2009) A proposal for a dimensional classification system based on the shared features of the DSM-IV anxiety and mood disorders: implications for assessment and treatment. Psychological Assessment 21: 256–271. 19719339 10.1037/a0016608 PMC2845450

[29] Musiat P, Hoffmann L, Schmidt U (2012) Personalised computerised feedback in E-mental health. Journal of Mental Health. 2012/02/10 ed. 10.3109/09638237.2011.648347 22315961

[30] Williams C (2009) Overcoming depression and low mood: a five areas approach. London: Hodder Arnold.

[31] Williams C, Wilson P, Morrison J, McMahon A, Walker A, et al (2013) Guided self-help cognitive behavioural therapy for depression in primary care: a randomised controlled trial. PLoS One 8: e52735. 23326352 10.1371/journal.pone.0052735 PMC3543408

[32] Sharpe M, Walker J, Williams C, Stone J, Cavanagh J, et al (2011) Guided self-help for functional (psychogenic) symptoms A randomized controlled efficacy trial. Neurology 77: 564–572. 21795652 10.1212/WNL.0b013e318228c0c7 PMC3149156

[33] Sánchez-Ortiz V, Munro C, Stahl D, House J, Startup H, et al (2011) A randomized controlled trial of internet-based cognitive-behavioural therapy for bulimia nervosa or related disorders in a student population. Psychological Medicine 41: 407. 20406523 10.1017/S0033291710000711

[34] Costa PT, McCrae RR (1992) Professional manual for the NEO PI-R. Odessa, FL: Psychological Assessment Resources.

[35] Frost RO, Marten P, Lahart C, Rosenblate R (1990) The Dimensions of Perfectionism. Cognitive therapy and research 14: 449–468.

[36] Stöber J (1998) The Frost Multidimensional Perfectionism Scale: More perfect with four (instead of six) dimensions. Personality and Individual Differences 24: 481–491.

[37] Woicik PA, Stewart SH, Pihl RO, Conrod PJ (2009) The substance use risk profile scale: A scale measuring traits linked to reinforcement-specific substance use profiles. Addictive Behaviors 34: 1042–1055. 19683400 10.1016/j.addbeh.2009.07.001

[38] Abramson LY, Metalsky GI, Alloy LB (1989) Hopelessness depression: A theory-based subtype of depression. Psychological Review 96: 358.

[39] Kroenke K, Spitzer RL, Williams JB (2001) The PHQ-9: validity of a brief depression severity measure. Journal of general internal medicine 16: 606–613. 11556941 10.1046/j.1525-1497.2001.016009606.x PMC1495268

[40] Spitzer RL, Kroenke K, Williams JB, Lowe B (2006) A brief measure for assessing generalized anxiety disorder: the GAD-7. Archives of Internal Medicine 166: 1092–1097. 16717171 10.1001/archinte.166.10.1092

[41] Saunders JB, Aasland OG, Babor TF, De La FuenteJR, Grant M (1993) Development of the Alcohol Use Disorders Identification Test (AUDIT): WHO Collaborative Project on Early Detection of Persons with Harmful Alcohol Consumption-II. Addiction 88: 791–804. 8329970 10.1111/j.1360-0443.1993.tb02093.x

[42] Fleming MF, Barry KL, MacDonald R (1991) The alcohol use disorders identification test (AUDIT) in a college sample. International Journal of the Addictions 26: 1173–1185. 1743817 10.3109/10826089109062153

[43] Stice E, Telch CF, Rizvi SL (2000) Development and validation of the Eating Disorder Diagnostic Scale: a brief self-report measure of anorexia, bulimia, and binge-eating disorder. Psychological Assessment 12: 123–131. 10887758 10.1037//1040-3590.12.2.123

[44] Rosenberg M (1965) Society and the adolescent self-image. Princeton, NJ: Princeton University Press.

[45] Martin-Albo J, Nuniez JL, Navarro JG, Grijalvo F (2007) The Rosenberg Self-Esteem Scale: translation and validation in university students. Spanish Journal of Psychology 10: 458–467. 17992972 10.1017/s1138741600006727

[46] The Whoqol Group (1998) Development of the World Health Organization WHOQOL-BREF quality of life assessment. The WHOQOL Group. Psychological medicine 28: 551–558. 9626712 10.1017/s0033291798006667

[47] The WHOQOL Group (1998) The World Health Organisation Quality of Life Assessment (WHOQOL)-Development and general psychometric properties. Social Science & Medicine 46: 1569–1585. 9672396 10.1016/s0277-9536(98)00009-4

[48] Kenardy J, McCafferty K, Rosa V (2003) Internet-delivered indicated prevention for anxiety disorders: A randomized controlled trial. Behavioural and Cognitive Psychotherapy 31: 279–289.

[49] Zabinski MF, Celio AA, Jacobs MJ, Manwaring J, Wilfley DE (2003) Internet-based prevention of eating disorders. European Eating Disorders Review 11: 183–197.

[50] Nebel MA (1995) Prevention of disordered eating among college women: A clinical intervention.

[51] Steinhardt M, Dolbier C (2007) Evaluation of a resilience intervention to enhance coping strategies and protective factors and decrease symptomatology. Journal of American College Health 56: 445–453. 10.3200/JACH.56.44.445-454 18316290

[52] Flett GL, Hewitt PL, De Rosa T (1996) Dimensions of perfectionism, psychosocial adjustment, and social skills. Personality and Individual Differences 20: 143–150.

[53] Pritchard ME, Wilson GS, Yamnitz B (2007) What predicts adjustment among college students? A longitudinal panel study. Journal of American College Health 56: 15–22. 17711821 10.3200/JACH.56.1.15-22

[54] Steele AL, Wade TD (2008) A randomised trial investigating guided self-help to reduce perfectionism and its impact on bulimia nervosa: a pilot study. Behaviour Research and Therapy 46: 1316–1323. 19007923 10.1016/j.brat.2008.09.006

[55] Maia BR, Soares MJ, Pereira AT, Marques M, Bos SC, et al (2011) Affective state dependence and relative trait stability of perfectionism in sleep disturbances. Revista Brasileira de Psiquiatria 33: 252–260. 21971778 10.1590/s1516-44462011000300008

[56] Mackenzie C, Gekoski W, Knox V (2006) Age, gender, and the underutilization of mental health services: the influence of help-seeking attitudes. Aging and Mental Health 10: 574–582. 17050086 10.1080/13607860600641200

[57] Palmqvist B, Carlbring P, Andersson G (2007) Internet-delivered treatments with or without therapist input: does the therapist factor have implications for efficacy and cost? Expert Review of Pharmacoeconomics and Outcomes Research 7: 291–297. 20528315 10.1586/14737167.7.3.291

[58] Craig P, Dieppe P, Macintyre S, Michie S, Nazareth I, et al (2008) Developing and evaluating complex interventions: the new Medical Research Council guidance. BMJ 337: 979–983. 10.1136/bmj.a1655 PMC2769032 18824488

[59] Melville KM, Casey LM, Kavanagh DJ (2010) Dropout from Internet-based treatment for psychological disorders. British Journal of Clinical Psychology 49: 455–471. 19799804 10.1348/014466509X472138

[60] Powell J, Hamborg T, Stallard N, Burls A, McSorley J, et al. (2013) Effectiveness of a web-based cognitive-behavioral tool to improve mental well-being in the general population: randomized controlled trial. Journal of Medical Internet Research 15.. 10.2196/jmir.2240 PMC3636304 23302475

