# Supplementary material for: Targeted Prevention of Common Mental Health Disorders in University Students: Randomised Controlled Trial of a Transdiagnostic Trait-Focused Web-Based Intervention
Source: PLoS One. 2014 Apr 15;9(4):e93621. doi: 10.1371/journal.pone.0093621 (PMC3988039; doi:10.1371/journal.pone.0093621)
Supplement: Protocol S1 — Trial protocol. (PDF) [file pone.0093621.s001.pdf]

STUDY PROTOCOL SUBMITTED THE KING'S COLLEGE RESEARCH ETHICS SUB-COMMITTEE

Trait focused internet-based prevention of common mental health problems in university students

Primary investigator:
Peter Musiat, Section of Eating Disorders, Psychological Medicine, Institute of Psychiatry

Email: peter.musiat@kcl.ac.uk

Telephone number: 020 7848 0183

Address: Section of Eating Disorders, PO-Box 059, Institute of Psychiatry, King's College London, De Crespigny Park, London, SE5 8AF

Name of Supervisor: Ulrike Schmidt

Funder: National Institute for Health Research (NIHR) Biomedical Research Centre for Mental Health at the South London and Maudsley NHS Foundation Trust (SLaM) and Institute of Psychiatry, King's College London

1. Background and aims

The aim of the proposed study is to investigate the efficacy and feasibility of an internet-based trait focused prevention programme for common mental health problems in university students.

The transition from school to university is associated with a rise in mental health problems. Up to 15% of students report moderate or severe levels of depression and about 20% of students report moderate or severe anxiety levels.[1] Up to 10% of female students report a current eating disorder. Furthermore, 24% of female and 40% of male student exceed safe drinking limits.[2] Research has identified a variety of personality risk factors for eating disorders (ED), substance abuse (SA) and depression (D) and there is significant overlap between the three disorders. Existing prevalence programmes usually focus only on one disorder (depression[3], eating disorders[4], alcohol misuse[5]) and proved most promising, if they target people at high risk.[6]

In a previous study on personality risk factors and well-being (PNM/08/09-142), personality risk factors were identified that allow identifying students who are more likely to be at risk for common mental health problems. Data from that study suggested that high levels of dysfunctional perfectionism, high trait anxiety and low self-esteem are associated with reduced well-being in university students. A qualitative study (PNM0910-80) investigated students' needs in terms psychological well-being and how students can be supported with those needs. The findings of both studies informed the development of the prevention programme.

An multimedia online-based prevention package for students was developed to target personality risk factors that are associated with the onset of common mental health problems such as depression, alcohol and substance abuse, eating disorders, and anxiety disorders.

In a randomised control trial, students are assessed on personality variables and measures of psychological and physical well-being and randomised into two groups, in which they either receive a personality targeted prevention intervention (prevention group) or other helpful information relevant for student living (control group). It is hypothesised that in

comparison with helpful information on student living the prevention intervention improves the mental health of students at high risk for developing mental disorders on dimension such as stress, anxiety, depression or quality of life.

References:

1 Webb, E., et al., Alcohol and drug use in UK university students. Lancet, 1996. 348(9032): p. 922-5.
2 Sell, L. and P. Robson, Perceptions of College Life, Emotional Well-Being and Patterns of Drug and Alcohol Use among Oxford Undergraduates. Oxford Review of Education, 1998. 24(2): p. 235-243.
3 Seligman, M.E.P., P. Schulman, and A.M. Tryon, Group prevention of depression and anxiety symptoms. Behaviour Research and Therapy, 2007. 45(6): p. 1111-1126.
4 Taylor, C.B., et al., Prevention of eating disorders in at-risk college-age women. Archives of General Psychiatry, 2006. 63(8): p. 881-888.
5 Conrod, P.J., et al., Efficacy of cognitive-behavioral interventions targeting personality risk factors for youth alcohol misuse. Journal of Clinical Child and Adolescent Psychology, 2006. 35(4): p. 550-563.
6 Stice, E. and H. Shaw, Eating disorder prevention programs: A meta-analytic review. Psychological Bulletin, 2004. 130(2): p. 206-227.

2. Methods

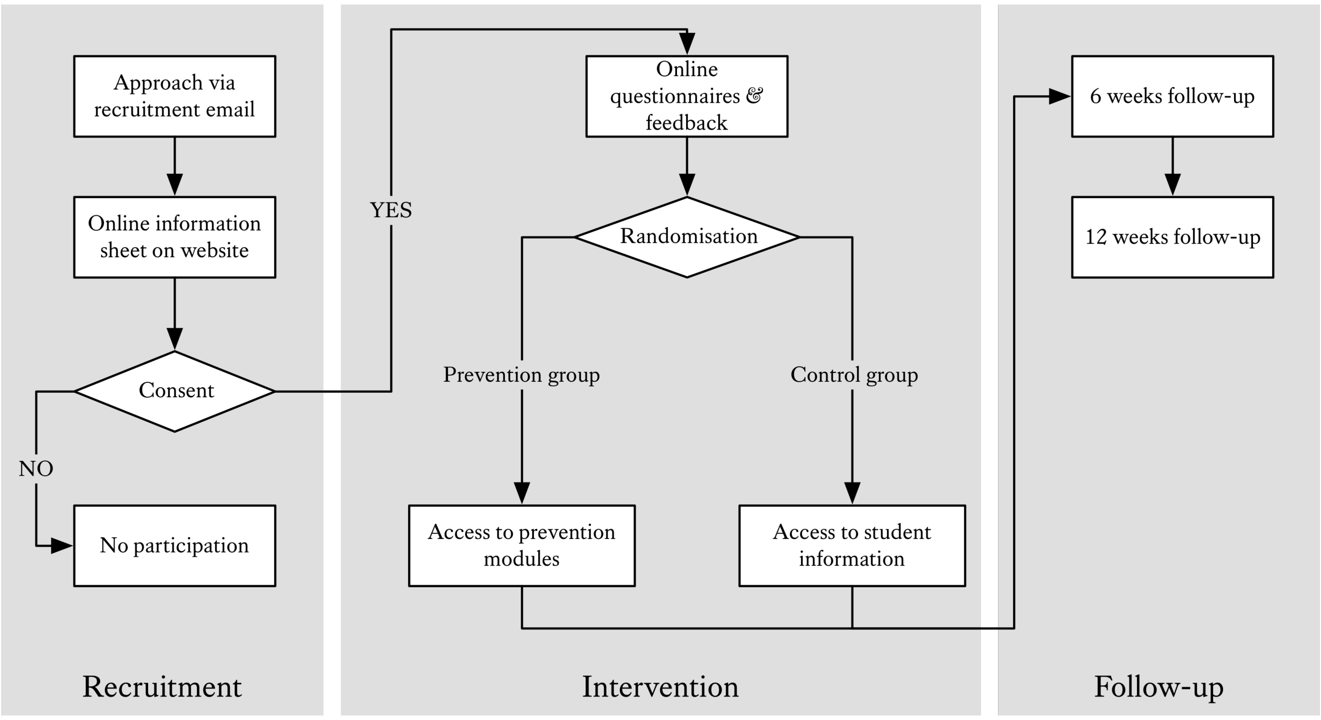

<u>Recruitment</u>

Students will be invited to participate in the research with a recruitment email sent out to undergraduate and postgraduate students of KCL, and other universities (upon receiving ethical approval from these universities). The email contains a link to the project website on which students can get more information about the project and participate. The project website will also be advertised by posters on public locations (e.g. university campus). If possible, the site project may be part of an article in the King's student magazine "roar" or of a feature on KCL-radio.

To participate, students have to create an online account on the page to gain access to the questionnaires and the site content. To be able to create the profile, students have to read the online information sheet and give consent. Students then can create an account with a freely chosen username.

<u>Baseline Questionnaires and Intervention</u>

After creating their account, students will be asked to complete the questionnaires for the first time. At Baseline, students will be asked to complete the following questionnaires:

- SURPS: The Substance Use Risk Profile Scale is a 23-item self-report measure to assess personality risk factors for substance abuse namely anxiety sensitivity, sensation seeking, hopelessness and impulsivity.
- AUDIT: The Alcohol Use Disorders Identification Test is a 10-item self-report measure to assess drinking behaviour and quantity.
- MPS-F The Frost Multidimensional Perfectionism Scale is a self-report measure to assess different aspects of perfectionism. For this study, 20 items of this questionnaire are included.
- EDDS: The Eating Disorders Diagnostics scale is a self-report questionnaire to assess the criteria for eating disorders according to the DSM-IV. It has 26 items with different rating scales.
- WHOQOL: The Quality Of Life questionnaire from the World Health Organisation is a global measure for quality of life on 4 different domains. These cover physical health, psychological health, social relationships, and environment on 26 items.
- NEO-FFI: the Neo-FFI is a 60-item self-report measure to assess personality on the dimensions neuroticism, extraversion, openness, conscientiousness and agreeableness.
- RSE: The Rosenberg Self-Esteem scale is a 10-item self-report questionnaire to assess self-esteem.
- PHQ9: The Patient Health Questionnaire is a self-report questionnaire to assess the severity of depression symptoms. I Has 9 items and participants have to indicate how often they have experienced each symptom within the last 2 weeks on a 4-step scale.
- GAD7: The General Anxiety Disorder scale is a brief self-report questionnaire to assess symptoms of general anxiety disorder. It consists of 7 items and participants have to indicate how often they have experienced symptoms within the past 2 weeks on a 4-step rating scale.

The total number of items is 205 and the baseline assessment takes about 20-40 minutes. This information is included in the information sheet. Students receive feedback on each questionnaire and subscale. The feedback includes

1. a description of the questionnaire and its subscales (e.g. "The WHOQOL is a questionnaire about your quality of life from the World Health Organisation. It assesses quality of life on four dimensions. These are: physical health, psychological health, social relationships and environment. Physical Health: This scale describes to what extent you suffer from medical problems and how they affect your everyday life")
2. a summary of their responses (e.g. "To some extent, your physical health can make it difficult for you do cope with everyday activities.")
3. a comparison of their response in relation to other students (e.g. "In comparison with other students at your age, your Psychological Health score is average")

The feedback does not contain any individual scores. After filling out the questionnaires and receiving feedback, students are randomised into two groups: (1) a control group and (2) a prevention group. The content of the website varies depending on which group students were randomised to. Students in the control group (1) get access to helpful information on studying (e.g. money saving tips, how to find accommodation in London, etc). Students in the prevention group (2) will gain access to the personality targeted prevention modules.

The prevention modules target personality risk factors for common mental health problems in students i.e. low self-esteem, high perfectionism and anxiety; and how to deal with difficult emotions. They are based on the 5-ares assessment approach (Williams, 2002) – an evidence based cognitive-behavioural intervention model – and were designed to help students with identifying cycles of unhelpful behaviour and breaking such cycles. Students in the prevention group can optionally download the content of the modules as PDF for offline use.

During the intervention stage, students of both groups will receive an email reminder to invite them to visit the site regularly.

Follow-up

After 6 weeks and 12 weeks students will be asked via email to log into their account to answer some of the questionnaires again. The following questionnaires are included in the follow-up assessment: AUDIT, DUDIT, MPS-F, EDDS, WHOQOL, IAPT, RSE, PHQ9 and GAD7 (for more information see above). The follow-up assessment includes approximately 120 items and takes about 10-25 minutes.

Data analysis

According to their results in the personality questionnaires at baseline, students will be grouped into two categories: students at *high risk* for mental health problems and students at *low risk*. Hence, a 2x2 design is created with two risk groups (*high/low*) and two intervention groups (*control/prevention*). Data will be analysed using a repeated measure MANOVA. Main effects are expected for groups and intervention type as well as an interaction effect between group and intervention type.

3. Participants

Number: 420
Selection criteria: Any undergraduate or postgraduate student studying in London is considered eligible to participate. There are no exclusion criteria.

How participants will be (i) identified and (ii) approached.

(i) The recruitment email will be sent out to undergraduate and postgraduate students studying at KCL, UCL, GS or SU. Each university usually has their email list for undergraduate/postgraduate students. Any student on this list is considered identified and will be approached.

(ii) Students will be approached via email (see above) or with posters at public places frequented by students, such as university message boards etc.

Location: Data assessment will be carried out by the web-intervention online. The project website is hosted by a UK webhosting company (simplewebhosting.co.uk). Their Servers are based in the UK and they are registered with the ICO (Registration Number: Z8908769) to ensure that data is handled in accordance with the Data Protection Act 1998. The evaluation and storage of the data takes place at the Eating Disorders Section at the Institute of Psychiatry (De Crespigny Park, London SE5 8AF), room M6/1/0020.

4. Ethical considerations

Informed consent:

To access the online-based questionnaires and the prevention programme, students have to create an account on the web page. Before creating this account, a web-version of the information sheet is presented and participants must tick a checkbox to indicate that they:

1. have understood the purpose and nature of the study, possible benefits or harm, and their right to withdraw at any given time, and
2. consent to taking part.

Written consent cannot be obtained in this project, as it is online based. The procedure above is to replace written consent and access to the questionnaires or prevention content is denied to anyone unable to tick the checkbox. As a preventative measure, the checkbox will active 1 minute after accessing the site to prevent students from just agreeing without having read the information sheet. No information about the student is collected prior to this consent procedure.

By showing the information sheet before the registration process, students have enough time to consider whether they want to participate.

No pressure to participate should arise from a possible relationship between the researcher and the student, as participants do not have to reveal their identity at any time or meet the researcher face-to-face.

Right of withdrawal:
Participants can withdraw from participation in the proposed study at any time, by not visiting the project website anymore. No contact between the participant and the involved researchers is necessary to withdraw.
Students can withdraw their data from the study until the time given in the information sheet by contacting the researcher and providing their online username.

Risk specific issues:

Inclusion of vulnerable individuals: The students invited to take part in this research project are not considered vulnerable as such. However, when working with a student population of that size, it is possible and likely few students are vulnerable or suffer from mental health problems. The project webpage does not offer any treatment and was not designed for student currently suffering from mental health problems. However, the site reminds the students of this and offers a list of helpful contacts for different problems (e.g. KCL student counselling, NHS direct, Samaritans, Beat (eating disorders), FRANK (alcohol problems)). Research suggests that students often use web-based intervention as a first step into treatment. One questionnaire includes an item on suicidal thoughts. Similar to the procedure from a previous study (PNM/08/09-142), if a participant reports having suicidal thoughts "almost every day" or "every day", the feedback for this questionnaire will automatically include the following message "Your answers in this questionnaire suggest that you have thoughts about hurting yourself or wanting to be dead. Although everyone experiences such thoughts from time to time, it might be a good idea to speak to someone about this. This could be a friend, a relative or your GP. You can also contact the phone numbers below at any time: NHS direct: 0845 4647, Samaritans: 08457 90 90 90"

Criminal disclosure: In response to the DUDIT questionnaire, students might disclose previous or current use of illegal substances. However, students do not have to answer to all questions of the questionnaire and data is anonymised.

Potential for distress or embarrassment: Participants are asked to fill out several questionnaires. These questionnaires cover personality, eating and drinking behaviour and general questions about mental health. Some participants might feel embarrassed or distressed about answering certain questions. The study deals with this issue in two ways.

- All assessments are internet-based and data is anonymised. Students can stop the assessment by closing their browser without having to contact a researcher or justifying their decision to withdraw.

Students have the option not to answer to all questions of each questionnaire. If they leave one or several items blank, they will be reminded and can decide not to answer.

Participants' knowledge about randomisation

Students will be informed in the information sheet that after filling out the questionnaire, they will gain access to the website, the content of which varies depending on the responses to the questionnaires. Students are not informed about the randomisation process. This procedure was chosen for three reasons:

- The site does not target students with mental health problems and does not offer treatment as such. Students in the control group therefore do not 'miss out' on an opportunity for improvement.
- In the control condition, the site still offers useful information related to student living. The difference in the content lies in the fact that content in the control condition does not target personality risk factors.
- To minimise a potential placebo-effect

Questionnaire feedback

Participants will receive feedback on the responses to the questionnaires. Some students might not understand the feedback. Therefore, it has to be designed in a comprehensive way. For that reason, each feedback element consists of three components. An explanation of what was measured with a particular questionnaire or scale, a summary of the participant's responses and (if applicable) a comparison of their response in relation to other students. The assessment does not include any performance measure. Therefore, there is no 'right' or 'wrong' in the answers. Any feedback does not evaluate the participants' responses but it designed to summarise them. Participants can contact the PI, if they have questions about the feedback. However, the PI cannot give individual feedback for reasons of anonymity.

Anonymisation

Although the proposed study is entirely web-based, it is necessary to collect information that under some circumstances may allow the identification of a particular student. Students are asked to provide a valid email address so that they can be sent email reminders and their voucher code after the completion of the study. Experience from previous studies has shown that many students user their university email, which contains their full name. However, students can provide any other valid email address. Furthermore, all data is anonymised. Emails will be stored in a separate encrypted SQL database along with the username of the student to avoid that the data can be immediately linked to a particular individual.

Benefits:

Regardless of which group students are randomised to, the website contains useful information, which was designed to help students with difficult aspects of student living. It is expected that after visiting the website, students may have reduced stress, depression and anxiety and show improved quality of life. Ultimately the intervention may prevent or delay the onset of common mental health problems in students.

By getting feedback on personality measures, participants learn about their strengths and weaknesses. Furthermore, by getting risk-specific feedback, students are able to identify early signs of problematic behaviour (such as problematic drinking or eating) and act on it. This could include changing their lifestyle in a positive way.

As the assessment is repeated after 6 weeks and 12 weeks, students get the opportunity to monitor and get feedback on their changes. This might be particularly interesting for students as they may not always be aware of lifestyle changes.

Students who complete all 3 assessments will also receive a small financial reward for their time and effort in form of a £15 Amazon voucher.

Students can request a copy of the final report after the study has been completed and written up.

Risks:
Students will receive feedback on the results of the completed questionnaires. The feedback includes a description of the measured scale or trait, a summary of the participant's responses as well as a comparison with other people at the same age. This might be distressing to some participants, especially if their self-perception differs significantly from the feedback. Students might not be aware of the fact, that some of their behaviours could indicate a risk or are harmful to them. The feedback is designed in a way that does not evaluate the participant's responses, but summarises them.

Some of the questionnaires include topics that might be distressing or embarrassing to the students (i.e. questions about drug use). However, students do not have to answer to any question unless they want to. The site contains link to health resources for students, such as KCL student support/counselling, an eating disorder charity, a helpline for alcohol users as well as NHS direct and the Samaritans. In case a student feel distressed in way that they would like to speak to someone about it, they can use these resources.

5. Data protection and confidentiality

As data collection is online based, all data are stored on the secure servers of a webhosting company (simplewebhosting.co.uk). Their Servers are based in the UK and they are registered with the ICO (Registration Number: Z8908769) to ensure that data is handled in accordance with the Data Protection Act 1998.
The web-hoster stores the data in encrypted SQL databases and only the involved researchers have access to it (password protected access). Compliance with the Data Protection Act has been confirmed and the King's legal team (email correspondence attached) gave further advice how to set up the contract with the company to ensure that.
Participants can logon to the project website with their username and a password. This login is procedure is protected with a secure connection protocol (SSL) to prevent anyone from obtaining these login details.
Information collected from the participants will be anonymised. Participants can be identified with their freely chosen online username. The only personal information collected from the students is the email address. This is necessary to contact students in case they have won a voucher or to send email reminders. However, students can provide an email, which does not reveal their identity. To ensure anonymity, the list of email addresses and usernames will be stored separately from questionnaire data.
Students will be informed about the handling of the data in the information sheet
For the evaluation of the data, databases will be downloaded to password protected PCs at the Institute of Psychiatry (De Crespigny Park, London SE5 8AF). Computers are located in the Eating Disorders Section in a shared office (room M6/1/0020) and only the PI (Peter Musiat) and the supervisor (Ulrike Schmidt) have access to the data.

Storage
Data will be stored:

1. for a maximum of 5 years, following the guidance from the Data Protection Act 1998
2. in an electronic format (e.g. SPSS data file)
3. on password protected PCs at the Eating Disorders Section (room M6/1/0020) at the Institute of Psychiatry (De Crespigny Park, London SE5 8AF).
